# Diagnostic Impact of Fetal MRI in 556 Fetuses: Where It Adds Value Beyond Ultrasound

**DOI:** 10.3390/jcm14196690

**Published:** 2025-09-23

**Authors:** Zübeyde Emiralioğlu Çakır, Hakan Golbasi, Raziye Torun, Ceren Sağlam, İlayda Gercik Arzık, Hale Ankara Aktaş, Sevim Tuncer Can, İlknur Toka, İlker Uçar, Fatma Ceren Sarıoğlu, Atalay Ekin

**Affiliations:** 1 Department of Perinatology, Izmir City Hospital, Izmir 35510, Turkey; drhkngolbasi@gmail.com (H.G.); drraziyetorun@gmail.com (R.T.); drcerensaglam@yahoo.com (C.S.); ilgercik@gmail.com (İ.G.A.); haleankara@gmail.com (H.A.A.); drsevimtuncer@hotmail.com (S.T.C.); drilknurtoka@gmail.com (İ.T.); atalayekin@hotmail.com (A.E.); 2 Department of Perinatology, Izmir Tepecik Training and Research Hospital, Izmir 35020, Turkey; ucar.ilker@yahoo.com; 3 Departmant of Radiology, Division of Pediatric Radiology, Dokuz Eylul University School of Medicine, Izmir 35340, Turkey; drcerenunal@gmail.com

**Keywords:** fetal MRI, prenatal ultrasound, diagnostic concordance, fetal anomalies, CNS anomalies, non-CNS anomalies, gastrointestinal anomalies, diagnostic yield

## Abstract

**Objectives**: This study aimed to assess the diagnostic contribution of fetal MRI across different anatomical systems and evaluate its added value beyond prenatal ultrasonography. **Methods**: This retrospective cohort included 556 fetuses who underwent both prenatal ultrasound and fetal MRI in a single tertiary center. Cases were classified by anatomical system. The concordance between ultrasound and MRI findings, as well as additional or ruled-out findings identified by MRI, was analyzed. Statistical significance and clinical relevance were also evaluated. **Results**: Among the 556 cases, complete concordance between ultrasound and MRI findings was observed in 48.9%. MRI ruled out the initial diagnosis in 20.1% and revealed additional findings in 32% of cases. A total of 192 additional findings were identified, while 115 previously suspected anomalies were ruled out. The highest diagnostic contribution was observed in central nervous system (CNS) and gastrointestinal system (GIS) anomalies. Posterior fossa abnormalities and cystic or mass lesions were frequently detected as additional findings on MRI. In contrast, ultrasound alone was generally sufficient for evaluating genitourinary (GUS), thoracic, and vertebral anomalies. The overall diagnostic yield of MRI was higher in anatomically complex or sonographically ambiguous cases. **Conclusions**: Fetal MRI provides significant additional diagnostic value, particularly in CNS and GIS anomalies, by detecting additional findings, clarifying uncertain diagnoses, or excluding suspected anomalies. Its selective use may enhance both prenatal counseling and postnatal management.

## 1. Introduction

The evaluation of fetal anatomy is most commonly performed using ultrasound (US), a widely accessible and affordable modality that enables dynamic real-time imaging. As ultrasound technology has evolved, its capacity to identify structural fetal abnormalities in different organ systems has markedly increased [1]. Even though an experienced sonographer can overcome most limitations of US, some limitations, such as body mass index (BMI) of the mother, fetal lie or presentation, and advanced gestational age, may be difficult to overcome [2,3].

Its ability to provide high-resolution images allows for detailed assessment of fetal conditions [4,5,6]. On the other hand, for fetal MRI, economic feasibility continues to be a key concern shared by families, healthcare professionals, and health insurance authorities [7].

The detection of fetal anomalies is essential for parental counseling as well as prenatal and postnatal management [8,9,10]. In cases involving multiple anomalies, the likelihood of underlying syndromic or chromosomal disorders is significantly increased, which may substantially influence clinical decision-making and management strategies [11,12,13].

Numerous studies have evaluated the diagnostic performance of fetal MRI in specific systems, mostly the central nervous system (CNS). Even when multiple anomalies are included, the analysis is typically restricted to the system initially indicated for MRI, overlooking incidental or additional findings in other regions [7,14].

Large-scale, single-center studies comparing ultrasound and MRI findings across a wide range of fetal anomalies, including multi-system involvement, are limited. This study aims to address this gap by evaluating diagnostic concordance, additional or ruled-out findings, and system-specific contributions across both CNS and non-CNS anomalies. It also seeks to determine which pregnancies benefit most from fetal MRI, in order to optimize its use in prenatal diagnosis and clinical management while avoiding unnecessary imaging.

## 2. Materials and Methods

### 2.1. Study Design and Population

This retrospective study included all fetal MRI examinations performed between January 2018 and September 2021. All pregnant women who underwent MRI due to suspected fetal anomalies from the first trimester onwards were included. Ultrasound examinations were performed between 12 and 38 weeks of gestation, and MRI examinations between 14 and 39 weeks, as shown in Table 1. 

**Figure 1:**
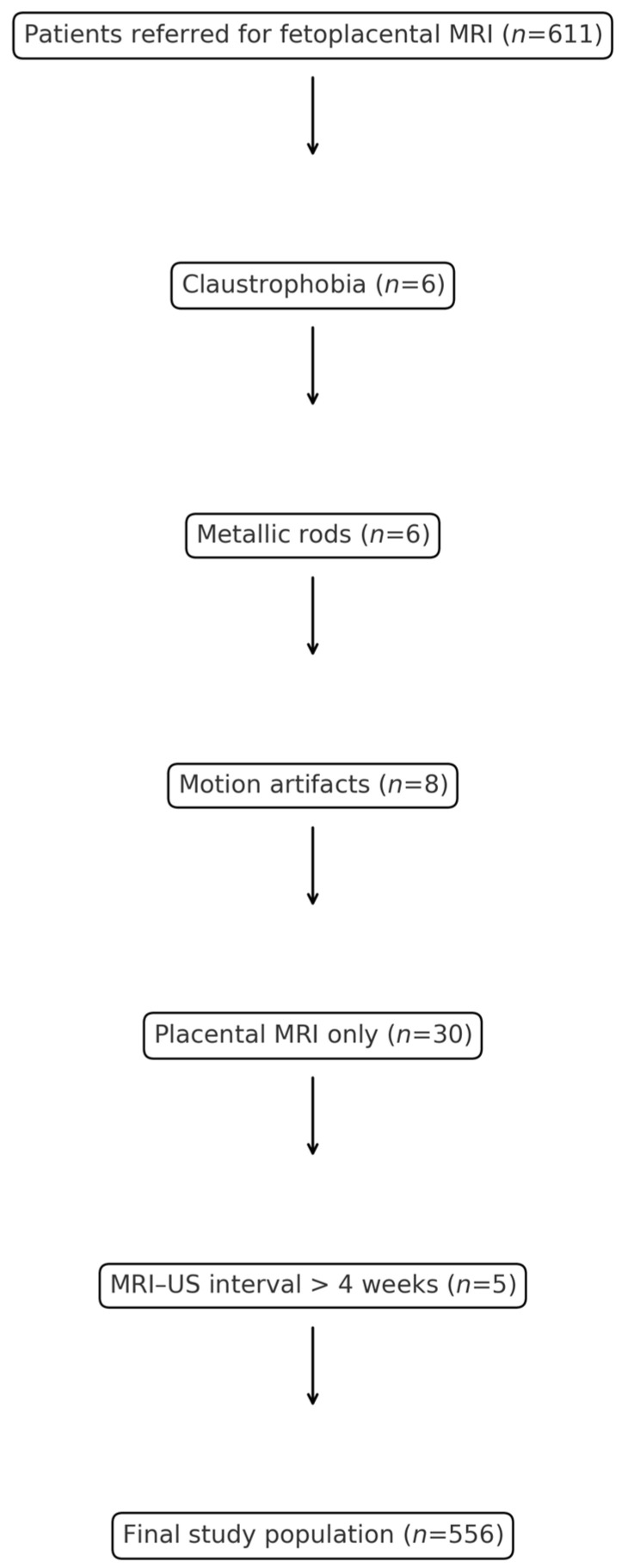
Flow chart of patient selection for the study. A total of 611 patients were referred for fetoplacental MRI. Exclusions included claustrophobia (*n* = 6), metallic rods (*n* = 6), motion artifacts (*n* = 8), placental MRI only (*n* = 30), and MRI–US interval > 4 weeks (*n* = 5). The final study population comprised 556 patients.

A total of 611 patients were referred for fetoplacental MRI. Of these, 6 examinations could not be performed due to claustrophobia, 6 patients were excluded because of metallic implants, and 8 were excluded owing to motion artifacts that precluded diagnostic-quality imaging. No patients were excluded because of high maternal BMI. Thus, 591 cases underwent MRI for either fetal or placental indications. For this study, only cases with fetal indications were included. After excluding 30 cases with isolated placental indications and 5 cases with an MRI–US interval longer than 4 weeks, the final study population comprised 556 fetuses, as illustrated in Figure 1. The study received approval from the Ethics Committee of İzmir Tepecik Research and Training Hospital (2022/10-26) and was conducted in accordance with the Declaration of Helsinki. As the study was retrospective, no study-specific informed consent was required. However, in line with institutional policy, written informed consent is routinely obtained from pregnant women prior to MRI procedures.

### 2.2. Ultrasound Imaging

All US examinations were conducted by experienced specialists in maternal-fetal medicine using a Samsung HS70A ultrasound system, equipped with CA1-7A transabdominal and V5-9 volumetric endocavity probes (Samsung Medison, Seoul, Republic of Korea). When necessary, fetal position was optimized by either waiting or re-evaluating the assessment using both transabdominal and transvaginal approaches. All US procedures were carried out in accordance with the guidelines of the International Society of Ultrasound in Obstetrics and Gynecology (ISUOG) [15,16,17,18,19,20] which included:

**Cranial and spinal:** cranial shape, cavum septi pellucidi, falx cerebri, thalami, cerebral hemispheres, ventricular system, cerebellum, cisterna magna, and vertebral column integrity in longitudinal and axial planes for anomalies such as spina bifida, sacral agenesis, or meningocele.

**Facial and neck:** orbits, ocular bulbs, midsagittal facial profile, nasal bone, lips, and neck masses (e.g., cystic hygroma).

**Thoracic:** chest shape and size, cardiac position, four-chamber view, left ventricular outflow tract (LVOT), aortic and pulmonary outflow tracts, and three-vessel views for great vessel relationships.

**Abdominal and urinary:** diaphragm continuity, stomach, bowel (dilatation or echogenicity), gallbladder, kidneys, bladder, and umbilical cord insertion.

All pregnancies referred due to fetal anomalies were first evaluated with a targeted ultrasound. Following this, a fetal anomaly screening was performed independently of gestational age to detect any potentially related anomalies. However, this anatomical survey was more limited after the mid-trimester. A detailed neurosonographic examination was performed in all cases, in line with the ISUOG guidelines [21,22,23].

### 2.3. Fetal MRI Acquisition

MRI was performed on a 1.5-T Siemens Aera scanner (Siemens Healthineers, Erlangen, Germany) using a six-channel body coil, following ISUOG fetal MRI guidelines [24]. Scanning was performed in supine or lateral decubitus positions without sedation or contrast agents. Standard sequences included T2-HASTE and TruFISP (4 mm slice thickness). T1-FLASH was added when clinically indicated, and diffusion-weighted imaging (DWI) was obtained in all cases, with B0 images used for hemorrhage detection if needed. Optional MRCP or cine sequences were performed to assess swallowing or limb motion. All scans were interpreted by an experienced pediatric radiologist.

### 2.4. Classification of Fetal Anomalies

MRI indications were determined by prior ultrasound examination and systematically categorized by anatomical region, including: CNS, vertebral, gastrointestinal system (GIS), genitourinary system (GUS), musculoskeletal, facial/neck, thoracic, undetermined abdominal cysts (UAC), cardiac, and unclassified anomalies (such as hydrops fetalis, fetal ascites, and perineal masses). Abdominal cysts suspected to originate from GIS or GUS were classified accordingly, whereas those with unknown origin were designated as undetermined abdominal cysts. MRI findings were similarly classified by system and analyzed separately as additional or ruled-out findings.

In each case, additional and ruled-out findings were documented independently. Cases in which none of these categories applied, and MRI findings were entirely consistent with US results, were defined as fully concordant. If only partial overlap existed between ultrasound and MRI findings, the case was categorized as partially concordant. When MRI was unable to clearly characterize a suspected anomaly, the findings were considered suspicious.

Diagnostic criteria for anomalies were consistent between ultrasound and MRI. Ventriculomegaly was defined as an atrial width of ≥10 mm measured in the axial transventricular plane. Pelviectasis was defined as a renal pelvis diameter ≥ 4 mm in the second trimester and ≥7 mm in the third trimester. Mega cisterna magna was defined as a cisterna magna width > 10 mm in the mid-sagittal or axial transcerebellar plane. The same cut-offs and measurement planes were applied in both imaging modalities.

### 2.5. Statistical Analysis

Descriptive statistics for the data were reported as mean, standard deviation, minimum, maximum, frequency, and percentage. For proportions, 95% confidence intervals were calculated using the Wilson method. Associations between categorical variables were analyzed using the Pearson Chi-square test or Fisher’s Exact Test when appropriate.

All statistical analyses were performed using IBM SPSS Statistics for Windows, Version 25.0 (IBM Corp., Armonk, NY, USA). A significance level of *p* < 0.05 was considered statistically significant.

## 3. Results

A total of 556 pregnant women with fetal anomalies detected on ultrasound were included. The mean maternal age was 27.6 ± 5.69 years, and the mean gestational ages at ultrasonography and MRI were 24.8 ± 5.37 and 25.92 ± 5.22 weeks, respectively. The average interval between USG and MRI was 1.09 weeks. Of all pregnancies, 98% were singleton and 2% were twin gestations (Table 1). Isolated anomalies were observed in 93.9% of cases, whereas multisystem involvement was detected in 6.1% on US.

US identified CNS anomalies as the most common indication (52.0%, 95% CI: 47.8–56.1), followed by GUS anomalies (12.4%, 95% CI: 9.9–15.4) and GIS anomalies (8.3%, 95% CI: 6.3–10.9). The distribution by system is shown in Table 2.

The distribution of cases in which the initial ultrasound-based suspicion either persisted or was partially confirmed after MRI varied across anatomical systems.

In UAC cases, the suspected diagnosis remained unchanged in 6 patients (21.4%, 95% CI: 10.2–39.5), while in other systems, such persistence was less frequent. Partial compatibility between US and MRI findings was observed in 53.6% of UAC cases (95% CI: 35.8–70.5; *n* = 15) and 50% of face and neck anomaly cases (95% CI: 18.8–81.2; *n* = 3), as summarized in Table 3.

MRI findings were fully concordant with ultrasound in 48.9% of cases (95% CI: 44.8–53.1), while 20.1% of suspected diagnoses were ruled out (95% CI: 17.0–23.7). Additional findings were observed in 32.0% 32.0% (95% CI: 28.3–36.0). The most frequent additional findings were identified in patients who underwent MRI for UAC, CNS, and GIS indications.

Among CNS anomalies(*n* = 289), MRI confirmed the US findings in 43.3% of cases (95% CI: 37.7–49.0; *n* = 125), which was relatively low compared to other systems (*p* = 0.005). The suspected diagnosis was ruled out in 23.5% of cases (95% CI: 19.0–28.7), and additional findings were identified in 34.6% (95% CI: 29.4–40.3). Of these, 9 patients (9%) had anomalies involving both CNS and non-CNS systems, while 7 patients (7%) had findings limited to non-CNS systems. The remaining 88 patients (88%) had additional findings confined to the CNS.

Vertebral anomalies (*n* = 42) demonstrated the highest rate of full concordance (73.8%, 95% CI: 58.9–84.7; *n* = 31) and the lowest rate of diagnostic change (*p* < 0.001). MRI ruled out vertebral anomalies in 4.8% of pregnancies (95% CI: 1.3–15.8), which was significantly lower compared with the exclusion rates observed in other systems (*p* = 0.010).

In GIS anomalies (*n* = 46), the rates of full concordance and exclusion were both 34.8% (95% CI: 22.7–49.2), with statistical significance for full concordance (*p* = 0.045) and exclusion (*p* = 0.010), respectively. In contrast, MRI revealed additional findings in 16 patients (34.8%, 95% CI: 22.7–49.2) evaluated for suspected GIS anomalies, including 5 cases (31.3%) with non-GIS anomalies, 1 case (6.3%) with both GIS and non-GIS findings, and 10 cases (62.5%) confined to the GIS.

Among GUS anomalies (*n* = 69), MRI and ultrasound findings were fully concordant in 69.6% of cases (95% CI: 57.9–79.2; *n* = 48). Additional findings were observed in only 18.8% of cases (95% CI: 11.4–29.6; *n* = 13). Both rates were significantly lower compared to other systems (*p* < 0.001 and *p* = 0.012, respectively).

In cases with UAC, the concordance rate was low (25.0%, 95% CI: 12.7–44.3; *n* = 7, *p* = 0.009), and MRI provided additional diagnostic information in 53.6% of cases (95% CI: 35.8–70.5; *n* = 15, *p* = 0.012. Among these, the cystic lesions were attributed to the GIS in 9 cases and to the ovary in 7 cases. No additional anomalies were detected in systems other than the primary cyst in these patients.

In cases with cardiac anomalies, MRI did not identify any structural cardiac abnormalities. In only one case with tetralogy of Fallot, MRI revealed an additional finding of unilateral mild ventriculomegaly.

For thoracic anomalies, MRI was fully concordant with US in 63.6% of cases (95% CI: 46.6–77.8) and ruled out the suspected diagnosis in only 6.1% (95% CI: 1.7–19.6, *p* = 0.038). A detailed breakdown by system is presented in Table 4.

**Figure 2:**
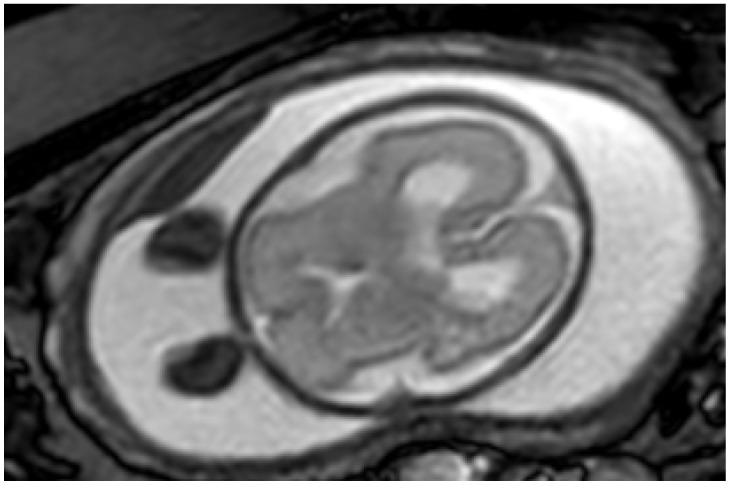
Axial MR image of a fetus at 25 weeks of gestation demonstrating bilateral mild ventriculomegaly.

Table 5 presents the distribution of additional findings identified by MRI and the US findings that were ruled out across all systems. The CNS accounted for the highest number of additional findings (*n* = 114). Posterior fossa anomalies and ventriculomegaly were frequently both newly identified and ruled out by MRI. An illustrative example of ventriculomegaly on MRI is shown in Figure 2.

Among posterior fossa anomalies, aqueductal stenosis was the most commonly added finding. Among the CNS findings ruled out by MRI, microcephaly and cavum septum pellucidum anomalies were the most common after posterior fossa anomalies and ventriculomegaly. In the vertebral system, closed neural tube defects were both the most frequently added and ruled-out findings.

**Figure 3:**
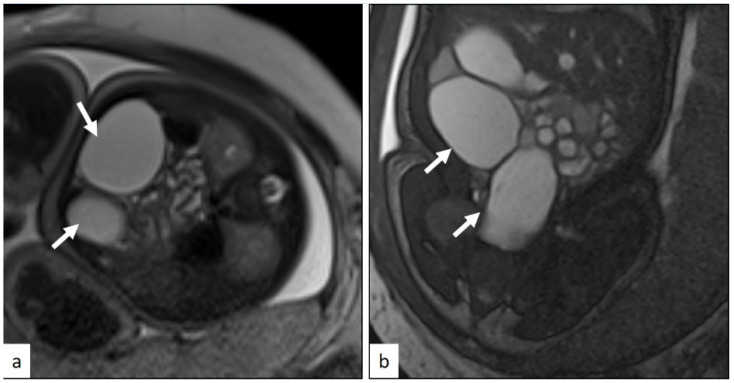
TruFISP axial (**a**) and coronal (**b**) planes MRI images demonstrate thin-walled cystic lesions in the midline and left side (arrows). There was no bowel obstruction. Kidneys were normal. The diagnosis was thought to be ovarian cysts. Postnatal diagnosis confirmed the ovarian cysts.

MRI identified 25 GIS anomalies, most of which were related to cyst characterization—primarily hepatic tumors—and bowel obstruction. It also ruled out 18 suspected findings, most commonly duodenal atresia and colonic dilatation. In the GUS group, MRI revealed 33 anomalies and ruled out 12. Ovarian cyst characterization was the most frequently added finding, while horseshoe kidney was the most commonly ruled out. An illustrative case is shown in Figure 3.

In the face and neck region, MRI detected 18 anomalies and ruled out 4. Hypertelorism was the most frequent anomaly in both categories. No additional findings were reported for abdominal cysts or skeletal anomalies. Except for a single case of craniosynostosis, MRI did not ruled out any skeletal or cardiac abnormalities.

Because more than one anomaly could be identified as an additional finding or ruled out in a single case, the total number of findings does not directly correspond to the number of fetuses with additional or ruled-out anomalies.

## 4. Discusion

In this study encompassing a broad spectrum of fetal anomalies, all US and MRI examinations were performed at a single institution. The most common indications for fetal MRI were CNS and GUS anomalies. While US and MRI findings were fully concordant in 48.9% of cases, MRI ruled out the initial diagnosis in 20.1% and revealed additional findings in 32% of patients. In total, MRI identified 208 additional findings and ruled out 115 previously suspected anomalies.

Among all fetal systems evaluated, MRI had a particularly high diagnostic impact in CNS anomalies. Previous large multicenter studies have consistently demonstrated the superior diagnostic accuracy of in-utero MRI compared with ultrasound (93% vs. 68%; difference 25%, 95% CI 21–29) [25]. In those cohorts, MRI provided additional diagnostic information in nearly half of the cases and modified prognostic assessment in approximately 20% and clinical management was altered in over one-third of cases [25]. In our study, the concordance with US was relatively low (43.3%, *p* = 0.005), with additional findings detected in 34.6% of cases, while the suspected diagnosis was ruled out in only 23.5% of cases (*p* = 0.038). These results are in line with previous studies highlighting the diagnostic value of fetal MRI in CNS evaluation [1,4,6,25]. Reported US–MRI concordance rates vary between 49.3% and 86.5%, while our rate was notably lower [14,26,27]. MRI’s contribution has varied in previous studies, ranging from 4.4 to 73% (in the absence of neurosonography), with intermediate rates such as 16.9% and 35% diagnostic revision reported [14,25,26,28,29] Although all patients underwent neurosonography, the low concordance in our study may be due to methodological differences. Unlike previous studies limited to CNS findings, MRI was requested primarily for challenging or equivocal cases in our clinic, and we also included extracranial anomalies detected in CNS-indicated MRIs. Moreover, the MRI examinations were interpreted by radiologists highly specialized in fetal neuroimaging, which likely enhanced the detection of subtle abnormalities and provided a broader perspective on MRI’s diagnostic utility in prenatal evaluation.

In our study, the most frequently added CNS findings by MRI were posterior fossa anomalies, supporting previous reports that emphasize MRI’s advantage in evaluating this region due to its ability to provide multiplanar imaging [29,30,31]. Aqueductal stenosis was the most common added anomaly in our cohort, highlighting MRI’s ability to detect subtle obstructive lesions often missed on US. Ventriculomegaly (VM) was the most commonly ruled out and the second most frequently added finding. In a series of 310 fetuses with isolated mild ventriculomegaly, Salomon et al. reported that ultrasound-detected ventriculomegaly was not confirmed by MRI in 23.2% of cases, while additional anomalies were identified by MRI in 19.4% of cases. No histological confirmation was performed in that cohort; however, only cases confirmed by prenatal MRI underwent postnatal neuroimaging and clinical follow-up [32]. These findings reinforce the limited concordance between US and MRI in VM evaluation.

In vertebral anomalies, the concordance between US and MRI was high (*p* < 0.001), while the rate of diagnosis being ruled out was significantly low (*p* = 0.010), suggesting limited added value of MRI in this subgroup. In contrast, Cai et al. reported a lower concordance rate (36.2%) but ruled out both open and closed neural tube defects, which were included in our series—potentially explaining the differences [33].

GUS anomalies represented one of the most common indications for fetal MRI in our study. To the best of our knowledge, our study represents the largest cohort in the literature comparing fetal MRI and US findings specifically in GUS anomalies. Concordance with US was high (69.6%, *p* < 0.001), and the rate of additional findings was relatively low (18.8%, *p* = 0.012), suggesting that US is generally sufficient for the evaluation of GUS anomalies. GUS anomalies. However, in selected cases, MRI may offer added value. Previous studies have reported higher MRI contribution rates (29% and 36%), but these involved smaller sample sizes and did not assess statistical significance or clinical impact [6,34]. In this context, our findings contribute robust data supporting the selective, case-based use of fetal MRI in GUS evaluation.

Similar to GUS anomalies, GIS anomalies in our study also constituted, to the best of our knowledge, the largest cohort to date comparing fetal MRI and US findings in this anomaly group, with a total of 46 cases. Among all systems, GIS anomalies demonstrated one of the lowest full concordance rates between US and MRI (34.8%, *p* = 0.045). MRI not only ruled out the suspected diagnosis in 34.8% of cases (*p* = 0.010) but also identified additional findings in another 34.8% of cases. In our study, MRI identified a total of 25 additional GIS findings across all patients. The most common of these were hepatic cyst characterization and the level of bowel obstruction, whereas dilated colon and duodenal atresia were frequently ruled out. These results underscore the utility of MRI in clarifying uncertain cystic structures and determining the site of intestinal obstruction—an advantage previously reported in smaller studies by Kul et al. and Manganaro et al. [6,35]. Our findings support the selective use of fetal MRI for the evaluation of GIS anomalies, especially in complex or inconclusive ultrasound cases, to enhance prenatal counseling and guide postnatal management.

In face and neck anomalies, MRI most often added or ruled out hypertelorism. Our findings highlight MRI’s value in detecting hypertelorism, a feature often overlooked in prior cleft-focused studies [36] Given its syndromic associations, hypertelorism on US should be interpreted with caution.

Thoracic pathologies showed higher concordance rates between MRI and US. Although the rate of additional findings was statistically lower compared to other systems, MRI provided further diagnostic information in only 24.2% of cases. Levine et al. reported a higher rate of additional findings (38%) in thoracic anomalies; however, clinical management was altered in only 8% of cases [37]. These results suggest that MRI may be useful in selected thoracic cases, although further studies are warranted in this area.

Abdominal cyst identification is critical for guiding postnatal management. Sherwood et al. reported that 29% of fetal abdominal cysts required surgical intervention [38]. In our cohort, MRI provided additional findings in 53.6% of cases (*p* = 0.012), in line with its significantly low concordance with US (*p* = 0.009), highlighting its value in characterizing uncertain lesions. Similarly, Hugele et al. reported an added value of 26.5%, increasing to 73.4% in upper abdominal cysts [39]. Notably, 20% of cases in our study remained inconclusive even after MRI—the highest rate among all systems—likely due to our strict inclusion criteria, which focused solely on cysts of unknown origin, unlike previous studies that included lesions with established associations. These findings support the selective use of fetal MRI in evaluating ambiguous abdominal cysts, although further studies are needed to clarify its impact on clinical decision-making.

To our knowledge, apart from a conference abstract by Herrera et al. (2013), which reported poor agreement between MRI and US (κ = 0.28) in fetuses with multiple anomalies, no full-text, system-based comparison has been published [40]. In our cohort, 34 fetuses had multiple anomalies, with a concordance rate of 58.8%. Although this rate indicates a moderate level of agreement between US and MRI, the difference did not reach statistical significance (*p* = 0.233).

Both ultrasound and MRI are considered safe imaging modalities during pregnancy and are generally well accepted by pregnant women [41,42,43]. Dütemeyer et al. reported that fetal MRI examinations were unsuccessful due to claustrophobia in 2.1% of cases [44]. In our cohort, this rate was 0.98%, further supporting the high acceptability of fetal MRI in pregnant women and aligning with the findings in the literature.

Although postnatal confirmation was not available in our cohort, previous studies have shown that MRI findings tend to correlate more closely with postnatal diagnoses than ultrasound, further underscoring its diagnostic reliability [25,45]. Moreover, there are also studies demonstrating that these contributions can directly influence clinical management decisions [25,46,47].

Taken together, when considering the specific contribution of fetal MRI—particularly in central nervous system (CNS) anomalies, gastrointestinal malformations, undefined abdominal cysts, and multiple anomalies—omitting MRI in selected cases may lead to incomplete, incorrect, or even over-diagnosis. Such limitations can influence clinicians’ approach to pregnancy management, including whether to offer termination of pregnancy, continuation of pregnancy, or prenatal referral to subspecialties. Moreover, ruling out overestimated anomalies can alleviate parental anxiety, while the detection of additional findings may significantly alter parental decision-making, such as acceptance of invasive karyotyping or even consideration of pregnancy termination. In addition, when the findings of MRI and ultrasound are concordant, the confirmation provided by an additional imaging modality may be reassuring for both families and clinicians.

On the other hand, US is the first-line imaging method. Also, in systems where US alone is sufficient, MRI may represent an additional cost. Moreover, in cases where fetal MRI is requested by clinicians but cannot be performed or is deemed non-diagnostic due to factors such as claustrophobia, motion artifacts, or metallic implants, families may experience concerns about having undergone an incomplete investigation.

MRI may still contribute to syndrome recognition by revealing additional findings, but further studies with larger sample sizes are necessary to clarify its clinical impact. The retrospective design, absence of postnatal or autopsy confirmation, absence of prenatal outcomes, and lack of data on clinical impact make it difficult to draw definitive conclusions or apply the results broadly. Although US examinations were interpreted by a limited number of experienced perinatologists and MRIs by experienced radiologists, interobserver variability may still exist in the evaluation of both imaging modalities. In addition, MRI was only performed in fetuses with US-detected anomalies, and sonographic findings were known to the radiologists, which may have introduced selection and interpretation bias despite the involvement of a limited number of experienced radiologists. Furthermore, no correction for multiple testing was applied, which represents another limitation and may have affected the interpretation of some results.

## 5. Conclusions

This study demonstrates that the diagnostic contribution of fetal MRI is limited in certain anomalies—such as those involving the genitourinary, thoracic, and vertebral systems—where US alone may be sufficient. In contrast, MRI adds significant value in the evaluation of CNS anomalies, GIS anomalies, abdominal cysts, and multisystem involvement.

It was particularly helpful in assessing CNS anomalies, especially posterior fossa malformations, with aqueductal stenosis being a frequent associated finding. MRI also proved useful in characterizing UAC and in identifying the level of bowel obstruction or excluding gastrointestinal obstructions.

Additionally, in fetal anomalies, MRI can reveal additional findings beyond the primary indication, offering a more comprehensive anatomical assessment and potentially aiding the recognition of syndromic or chromosomal anomalies.

Further studies are warranted to investigate the contribution of fetal MRI to both prenatal and postnatal pregnancy management, as well as its impact on parental anxiety and its cost-effectiveness across different healthcare systems and countries.

## Figures and Tables

**Table 1:** Demographic characteristics of the study population.

	Mean ± SD (Min–Max)
Age	27.60 ± 5.69 (18–45)
Gravida	2.11 ± 1.34 (0–10)
Parity	0.88 ± 0.98 (0–6)
Living children	0.83 ± 0.96 (0–6)
Abortions	0.31 ± 0.83 (0–9)
Gestational age at US (weeks)	24.8 ± 5.37 (12–38)
Gestational age at MRI (weeks)	25.92 ± 5.22 (14–39)
US-MRI interval (weeks)	1.09 ± 0.94 (0–4)
	*n* (%)
Singleton pregnancy	545 (98.0%)
Twin pregnancy	11 (2.0%)
Total number of patients	556 (100.0%)

This table summarizes the demographic and clinical characteristics of the study population. Continuous variables are presented as mean ± standard deviation (SD) with minimum–maximum values in parentheses, and categorical variables as number (percentage). **Abbreviations**: US: Ultrasonography, MRI: Magnetic Resonance Imaging.

**Table 2:** Distribution of fetal anomalies by system.

Anomalies	*n*	%	95% CI
CNS	289	52.0%	(47.8–56.1)
Vertebra	42	7.6%	(5.6–10.1)
GIS	46	8.3%	(6.3–10.9)
GUS	69	12.4%	(9.9–15.4)
Face and Neck	6	1.1%	(0.5–2.3)
UAC	28	5.0%	(3.5–7.2)
Cardiac	5	0.9%	(0.4–2.1)
Thorax	33	5.9%	(4.3–8.2)
UA	1	0.2%	(0.0–1.0)
Skeletal	3	0.5%	(0.2–1.6)
Multiple Anomalies	34	6.1%	(4.4–8.4)
Total	556	100.0%	

This table shows the distribution and 95% confidence intervals for each anomaly group. **Abbreviations:** CNS, central nervous system; GIS, gastrointestinal system; GUS, genitourinary system; UAC: Undetermined Abdominal Cysts; Unclassified Anomalies (UA); CI, confidence interval.

**Table 3:** Changes in findings after MRI.

Anomalies	Suspicious			Partially Concordant		
	*n*	%	95% CI	*n*	%	95% CI
CNS	2	0.7	(0.2–2.5)	93	32.2	(27.1–37.8)
Vertebra	0	0.0	(0.0–8.4)	9	21.4	(11.7–35.9)
GIS	3	6.5	(2.2–17.5)	11	23.9	(13.9–37.9)
GUS	0	0.0	(0.0–5.3)	11	15.9	(9.1–26.3)
Face and Neck	0	0.0	(0.0–39.0)	3	50.0	(18.8–81.2)
UAC	6	21.4	(10.2–39.5)	15	53.6	(35.8–70.5)
Cardiac	0	0.0	(0.0–43.4)	0	0.0	(0.0–43.4)
Thorax	2	6.1	(1.7–19.6)	9	27.3	(15.1–44.2)
UA	0	0.0	(0.0–79.3)	0	0.0	(0.0–79.3)
Skeletal	0	0.0	(0.0–56.1)	2	66.7	(20.8–93.9)
Multiple Anomalies	1	2.9	(0.5–14.9)	12	35.3	(21.5–52.1)
Total	14	2.5	(1.5–4.2)	165	29.7	(26.0–33.6)

This table summarizes the number and proportion of cases in which anomalies were reclassified as suspicious or partially concordant following MRI, together with 95% confidence intervals. **Abbreviations:** CNS, central nervous system; GIS, gastrointestinal system; GUS, genitourinary system; UAC: Undetermined Abdominal Cysts; Unclassified Anomalies (UA); CI, confidence interval.

**Table 4:** Diagnostic contribution of MRI.

	Fully Concordance			Ruled Out			Additional Finding		
Anomalies	*n* (%)	95% CI	*p* Value	*n* (%)	95% CI	*p* Value	*n* (%)	95% CI	*p* Value
CNS	125 (43.3)	(37.7–49.0)	**0.005**	68 (23.5)	(19.0–28.7)	**0.038**	100 (34.6)	(29.4–40.3)	0.174
Vertebra	31 (73.8)	(58.9–84.7)	**<0.001**	2 (4.8)	(1.3–15.8)	**0.010**	9 (21.4)	(11.7–35.9)	0.126
GIS	16 (34.8)	(22.7–49.2)	**0.045**	16 (34.8)	(22.7–49.2)	**0.010**	16 (34.8)	(22.7–49.2)	0.674
GUS	48 (69.6)	(57.9–79.2)	**<0.001**	10 (14.5)	(8.1–24.7)	0.211	13 (18.8)	(11.4–29.6)	**0.012**
F&N	2 (33.3)	(9.7–70.0)	0.443	0 (0.0)	(0.0–39.0)	0.606	3 (50.0)	(18.8–81.2)	0.342
UAC	7 (25.0)	(12.7–43.4)	**0.009**	4 (14.3)	(5.7–31.5)	0.428	15 (53.6)	(35.8–70.5)	**0.012**
Cardiac	1 (20.0)	(3.6–62.4)	0.194	0 (0.0)	(0.0–43.4)	0.589	1 (20.0)	(3.6–62.4)	0.563
Thorax	21 (63.6)	(46.6–77.8)	0.081	2 (6.1)	(1.7–19.6)	**0.038**	8 (24.2)	(12.8–41.0)	0.324
UA	1 (100.0)	(20.7–100.0)	NA	0 (0.0)	(0.0–79.3)	NA	0 (0.0)	(0.0–79.3)	NA
Skeletal	0 (0.0)	(0.0–56.1)	NA	1 (33.3)	(6.1–79.2)	NA	2 (66.7)	(20.8–93.9)	NA
MA	20 (58.8)	(42.2–73.6)	0.233	9 (26.5)	(14.6–43.1)	0.342	11 (32.4)	(19.1–49.2)	0.965
Total	272 (48.9)	(44.8–53.1)		112 (20.1)	(17.0–23.7)		178 (32.0)	(28.3–36.0)	

This table presents the proportion of cases with full concordance, ruled-out diagnoses, and additional findings after MRI, together with corresponding 95% confidence intervals and *p* values. **Abbreviations:** CNS, central nervous system; GIS, gastrointestinal system; GUS, genitourinary system; F&N, Face and Neck, UAC: Undetermined Abdominal Cysts; UA: Unclassified Anomalies; MA, Multiple Anomalies; NA, Not Available; CI, confidence interval.

**Table 5:** Additional and ruled-out findings on MRI.

	Additional Findings	*n*	%	95% CI	Ruled-Out Findings	*n*	%	95% CI
**CNS**	PF Anomalies	42	36.8	(28.6–46.0)	Ventriculomegaly	22	31	(21.4–42.5)
	* AS*	16	38.1	(25.0–53.2)	PF Anomalies	15	21.1	(13.2–32.0)
	* MCM*	10	23.8	(13.5–38.5)	* MCM*	7	46.7	(24.8–69.9)
	* Cerebellar Hypoplasia*	5	11.9	(5.2–25.0)	* Cerebellar Hypoplasia*	2	13.3	(3.7–37.9)
	* Walker–Warburg Syndrome*	4	9.5	(3.8–22.1)	* Vermian Hypoplasia*	5	33.3	(15.2–58.3)
	* Vermian Hypoplasia*	3	7.1	(2.5–19.0)	* Blake’s Pouch Cyst*	1	6.7	(1.2–29.8)
	* Blake’s Pouch Cyst*	3	7.1	(2.5–19.0)	Microcephaly	13	18.3	(11.0–28.8)
	* Joubert Syndrome*	1	2.4	(0.4–12.3)	CSP Anomalies/Agenesis	9	12.7	(6.8–22.4)
	Ventriculomegaly	33	28.9	(21.4–37.9)	Macrocephaly	7	9.9	(4.9–19.0)
	CC Agenesis/Hypoplasia/Dysgenesis	14	12.3	(7.5–19.6)	Cc Agenesis/Hypoplasia	4	5.6	(2.2–13.6)
	CDM	8	7	(3.6–13.2)	Occipital Encephalocele	1	1.4	(0.2–7.6)
	Midline Developmental Anomaly	5	4.4	(1.9–9.9)	**Total**	**71**	100	
	GMH	5	4.4	(1.9–9.9)				
	Dysmorphic CSP	2	1.8	(0.5–6.2)				
	Cystic Lesion Characterization (AC)	2	1.8	(0.5–6.2)				
	Pericallosal Lipoma	1	0.9	(0.2–4.8)				
	Hydranencephaly	1	0.9	(0.2–4.8)				
	Periventricular Cyst	1	0.9	(0.2–4.8)				
	**Total**	**114**	100					
**Vertebra**	Closed NTD	7	77.8	(45.3–93.7)	Closed NTD	2	100	(34.2–100.0)
	Spinal arachnoid cyst	1	11.1	(2.0–43.5)	**Total**	**2**	100	
	Kyphosis	1	11.1	(2.0–43.5)				
	**Total**	**9**	100					
**GIS**	Cyst Characterization	13	52	(33.5–70.0)	Colonic Dilatation	7	38.9	(20.3–61.4)
	* Hepatic Tumors*	6	46.2	(23.2–70.9)	Duodenal Atresia	6	33.3	(16.3–56.3)
	* Mesenteric Cyst*	4	30.8	(12.7–57.6)	Intra-Abdominal Calcification	2	11.1	(3.1–32.8)
	* Colonic duplication cyst*	3	23.1	(8.2–50.3)	Esophageal Atresia	2	11.1	(3.1–32.8)
	Level Of Bowel Obstruction	7	28	(14.3–47.6)	Hydropic Gallbladder	1	5.6	(1.0–25.8)
	Meconium Peritonitis	2	8	(2.2–25.0)	**Total**	**18**	100	
	Fokal Aganglionik Segment	1	4	(0.7–19.5)				
	Inguinal Hernia	1	4	(0.7–19.5)				
	Gallbladder Agenesis	1	4	(0.7–19.5)				
	**Total**	**25**	100					
**GUS**	Ovarian Cyst Characterization	16	48.5	(32.5–64.8)	Horseshoe Kidney	3	25	(8.9–53.2)
	Ureteropelvic Duplication	6	18.2	(8.6–34.4)	BRS↑	2	16.7	(4.7–44.8)
	Renal Agenesis/Hypoplasia	2	6.1	(1.7–19.6)	BKE↑	2	16.7	(4.7–44.8)
	Duplicated Collecting System	2	6.1	(1.7–19.6)	Duplicated Collecting System	1	8.3	(1.5–35.4)
	UB Ureteral Dilatation	2	6.1	(1.7–19.6)	Renal Agenesis	1	8.3	(1.5–35.4)
	Horseshoe Kidney	1	3	(0.5–15.3)	Renal Pyelectasis	1	8.3	(1.5–35.4)
	MCDK	1	3	(0.5–15.3)	Ambiguous genitalia	1	8.3	(1.5–35.4)
	Renal Cortical Cyst	1	3	(0.5–15.3)	Adrenal cyst	1	8.3	(1.5–35.4)
	Ectopic Kidney	1	3	(0.5–15.3)	**Total**	**12**	100	
	Bilateral Hydronephrosis	1	3	(0.5–15.3)				
	**Total**	**33**	100					
**Skeletal**		-			Craniosynostosis	1	100	(20.7–100.0)
	**Total**	**0**	0		**Total**	1	100	
**F&N**	Hypertelorism	10	55.6	(33.7–75.4)	Hypertelorism	3	75	(30.1–95.4)
	Increased Nuchal Thickness	3	16.7	(5.8–39.2)	Micrognathia	1	25	(4.6–69.9)
	Cleft Palate	1	5.6	(1.0–25.8)	**Total**	**4**	100	
	Cystic Lymphangioma	1	5.6	(1.0–25.8)				
	Branchial Cleft Cyst	1	5.6	(1.0–25.8)				
	Cleft Lip And Palate	1	5.6	(1.0–25.8)				
	Dysmorphic Face	1	5.6	(1.0–25.8)				
	**Total**	**18**	100					
**Thorax**	Mass Identification	4	57.1	(25.0–84.2)	BLE↑	2	66.7	(20.8–93.9)
	* Pulmonary Sequestration*	3	75	(30.1–95.4)	Enlarged Thymus	1	33.3	(6.1–79.2)
	* CCAM*	1	25	(4.6–69.9)	**Total**	**3**	100	
	Congenital Diaphragmatic Hernia	2	28.6	(8.2–64.1)				
	Congenital Lobar Emphysema	1	14.3	(2.6–51.3)				
	**Total**	**7**	100					
**UAC**	**Total**	**0**	0		**Total**	**4**	100	
**Cardiac**	Cardiomegaly	1	100	(20.7–100.0)		-		
	**Total**	**1**	100		**Total**	**0**	0	
	Body Stalk Anomaly	1	100	(20.7–100.0)		-		
**UA**	**Total**	**1**	100		**Total**	**0**	0	
	**Total**	208	100		**Total**	115	100	

This table presents the distribution of anomalies newly identified (additional findings) or ruled out by MRI, together with the corresponding numbers, percentages, and 95% confidence intervals. **Abbreviations**: AS: aqueductal stenosis, MCM: mega cisterna magna, CC: corpus callosum, CDM: cortical developmental malformations, GMH: germinal matrix hemorrhage, CSP: cavum septum pellucidum, AC: aqueductal stenosis, UB: unilateral or bilateral, NTD: neural tube defect, CDK: multicystic dysplastic kidney, CCAM: congenital cystic adenomatoid malformation, BRS↑: bilateral renal enlargement, BKE↑: bilateral increased kidney echogenicity, BLE↑: bilateral increased lung echogenicity.

## Data Availability

Data are contained within the article.

## References

[1] Santos X.M., Papanna R., Johnson A., Cass D.L., Olutoye O.O., Moise K.J., Belleza-Bascon B., Cassady C.I. (2010). The use of combined ultrasound and magnetic resonance imaging in the detection of fetal anomalies. Prenat. Diagn..

[2] Griffiths P.D., Bradburn M., Campbell M.J., Cooper C.L., Embleton N., Graham R., Hart A.R., Jarvis D., Kilby M.D., Lie M. (2019). MRI in the diagnosis of fetal developmental brain abnormalities: The MERIDIAN diagnostic accuracy study. Health Technol. Assess..

[3] Blondiaux E., Garel C. (2013). Fetal cerebral imaging—Ultrasound vs. MRI: An update. Acta Radiol..

[4] Frates M.C., Kumar A.J., Benson C.B., Ward V.L., Tempany C.M. (2004). Fetal anomalies: Comparison of MR imaging and US for diagnosis. Radiology.

[5] Breysem L., Bosmans H., Dymarkowski S., Van Schoubroeck D., Witters I., Deprest J., Demaerel P., Vanbeckevoort D., Vanhole C., Casaer P. (2003). The value of fast MR imaging as an adjunct to ultrasound in prenatal diagnosis. Eur. Radiol..

[6] Kul S., Korkmaz H.A.A., Cansu A., Dinc H., Ahmetoglu A., Guven S., Imamoglu M. (2012). Contribution of MRI to ultrasound in the diagnosis of fetal anomalies. J. Magn. Reson. Imaging.

[7] Adiyaman D., Öztekin Ö., Kuyucu M., Atakul B.K., Toklu G., Aykut İ., Yıldırım A.G.Ş., Özeren M., Öztekin D. (2022). Contribution of fetal magnetic resonance imaging in the evaluation of neurosonographically detected cases of isolated mild and moderate cerebral ventriculomegaly. J. Obstet. Gynaecol. Res..

[8] Edwards L., Hui L. (2018). First and second trimester screening for fetal structural anomalies. Semin. Fetal Neonatal Med..

[9] Yılmaz G.M., Demir S.C., Aykut S., Evrüke İ.C., Sucu M. (2025). Evaluation of prenatal and postnatal outcomes of fetuses with intrauterine cardiac anomalies: Tertiary center experience. Turk. J. Obstet. Gynecol..

[10] Gagnon A., Wilson R.D., Allen V.M., Audibert F., Blight C., Brock J.A., Désilets V.A., Johnson J.-A., Langlois S., Murphy-Kaulbeck L. (2009). Evaluation of Prenatally Diagnosed Structural Congenital Anomalies. J. Obstet. Gynaecol. Can..

[11] Morris J.K., Bergman J.E.H., Barisic I., Wellesley D., Tucker D., Limb E., Addor M.-C., Cavero-Carbonell C., Dias C.M., Draper E.S. (2024). Surveillance of multiple congenital anomalies; searching for new associations. Eur. J. Hum. Genet..

[12] Khoury M.J., Adams M.M., Rhodes P., Erickson J.D. (1987). Monitoring for multiple malformations in the detection of epidemics of birth defects. Teratology.

[13] Rizzo N., Pittalis M.C., Pilu G., Perolo A., Banzi C., Visentin A., Bovicelli L. (1996). Distribution of abnormal karyotypes among malformed fetuses detected by ultrasound throughout gestation. Prenat. Diagn..

[14] Moradi B., Zare Bidoki F., Azadbakht J., Shirazi M., Hashemi H., Hantooshzadeh S., Kazemi M.A., Shafiee M., Golezar M.H. (2024). Comparing the Diagnostic Yield of Antenatal Fetal Ultrasound, Neurosonography, and MRI for Detecting CNS Anomalies: A Prospective Study. Neurol. Lett..

[15] Performance of the Routine Mid-Trimester Fetal Ultrasound Scan. https://www.isuog.org/resource/isuog-practice-guidelines-updated-performance-of-the-routine-mid-trimester-fetal-ultrasound-scan.html.

[16] Updated ISUOG Practice Guidelines: Performance of 11-14-Week Ultrasound Scan. https://www.isuog.org/resource/updated-isuog-practice-guidelines-performance-of-11-14-week-ultrasound-scan.html.

[17] Updated ISUOG Practice Guidelines: Fetal Cardiac Screening. https://www.isuog.org/resource/updated-isuog-practice-guidelines-fetal-cardiac-screening.html.

[18] Salomon L.J., Alfirevic Z., Berghella V., Bilardo C., Hernandez-Andrade E., Johnsen S.L., Kalache K., Leung K., Malinger G., Munoz H. (2011). Practice guidelines for performance of the routine mid-trimester fetal ultrasound scan. Ultrasound Obstet. Gynecol..

[19] Salomon L.J., Alfirevic Z., Bilardo C.M., Chalouhi G.E., Ghi T., Kagan K.O., Lau T.K., Papageorghiou A.T., Raine-Fenning N.J., Stirnemann J. (2013). ISUOG practice guidelines: Performance of first-trimester fetal ultrasound scan. Ultrasound Obstet. Gynecol..

[20] Carvalho J., Allan L., Chaoui R., Copel J., DeVore G., Hecher K., Lee W., Munoz H., Paladini D., Tutschek B. (2013). ISUOG Practice Guidelines (updated): Sonographic screening examination of the fetal heart. Ultrasound Obstet. Gynecol..

[21] Paladini D., Malinger G., Monteagudo A., Pilu G., Timor-Tritsch I., Toi A. (2007). Sonographic examination of the fetal central nervous system: Guidelines for performing the “basic examination” and the “fetal neurosonogram”. Ultrasound Obstet. Gynecol..

[22] Malinger G., Paladini D., Haratz K.K., Monteagudo A., Pilu G.L., Timor-Tritsch I.E. (2020). ISUOG Practice Guidelines (updated): Sonographic examination of the fetal central nervous system. Part 1: Performance of screening examination and indications for targeted neurosonography. Ultrasound Obstet. Gynecol..

[23] Paladini D., Malinger G., Birnbaum R., Monteagudo A., Pilu G., Salomon L.J., Timor-Tritsch I.E. (2021). ISUOG Practice Guidelines (updated): Sonographic examination of the fetal central nervous system. Part 2: Performance of targeted neurosonography. Ultrasound Obstet. Gynecol..

[24] Prayer D., Malinger G., Brugger P.C., Cassady C., De Catte L., De Keersmaecker B., Fernandes G.L., Glanc P., Gonçalves L.F., Gruber G.M. (2017). ISUOG Practice Guidelines: Performance of fetal magnetic resonance imaging. Ultrasound Obstet. Gynecol..

[25] Nielsen B.W., Scott R.C. (2017). Brain abnormalities in fetuses: In-utero MRI versus ultrasound. Lancet.

[26] Demir S.S., Cagliyan E., Sarioglu F.C., Guleryuz H., Altunyurt S. (2022). Diagnosis of central nervous system abnormalities: Comparison of prenatal neurosonography and foetal magnetic resonance imaging findings. J. Obstet. Gynaecol..

[27] Paladini D., Quarantelli M., Sglavo G., Pastore G., Cavallaro A., D’Armiento M.R., Salvatore M., Nappi C. (2014). Accuracy of neurosonography and MRI in clinical management of fetuses referred with central nervous system abnormalities. Ultrasound Obstet. Gynecol..

[28] Kirtis E., Bulbul G.A., Kandemir H., Sanhal C.Y., Karaali K., Mendilcioglu I.I. (2024). Additive Effect of Fetal Magnetic Resonance Imaging to Prenatal Ultrasonography in Fetal Congenital Anomalies. Gynecol. Obstet. Reprod. Med..

[29] Whitby E.H., Paley M.N.J., Sprigg A., Rutter S., Davies N.P., Wilkinson I.D., Griffiths P. (2004). Comparison of ultrasound and magnetic resonance imaging in 100 singleton pregnancies with suspected brain abnormalities. BJOG.

[30] Miller E., Orman G., Huisman T.A.G.M. (2021). Fetal MRI assessment of posterior fossa anomalies: A review. J. Neuroimaging.

[31] Bowker R.M., Marathu K.K., Pharel M., Adepoju J.O., Vahedifard F., Adler S., Kocak M., Liu X., Byrd S.E. (2025). Utility of Biometric Measurements from Fetal Magnetic Resonance Imaging for Improved Antenatal Diagnosis of Dandy–Walker Spectrum Posterior Fossa Lesions. Diagnostics.

[32] Salomon L.J., Ouahba J., Delezoide A.L., Vuillard E., Oury J.F., Sebag G., Garel C. (2006). Third-trimester fetal MRI in isolated 10-to 12-mm ventriculomegaly: Is it worth it?. BJOG.

[33] Cai X., Chen X., Wei X., Liu W., Hou X., Gong T., Zhu J., Haacke E.M., Wang G. (2022). Use of magnetic resonance imaging in the diagnosis of fetal vertebral abnormalities in utero: A single-center retrospective cohort study. Quant. Imaging Med. Surg..

[34] Barseghyan K., Jackson H.A., Chmait R., De Filippo R.E., Miller D.A. (2008). Complementary roles of sonography and magnetic resonance imaging in the assessment of fetal urinary tract anomalies. J. Ultrasound Med..

[35] Manganaro L., Saldari M., Bernardo S., Vinci V., Aliberti C., Sollazzo P., Giancotti A., Capozza F., Porpora M.G., Cozzi D.A. (2015). Role of magnetic resonance imaging in the prenatal diagnosis of gastrointestinal fetal anomalies. Radiol. Medica.

[36] Arangio P., Manganaro L., Pacifici A., Basile E., Cascone P. (2013). Importance of fetal MRI in evaluation of craniofacial deformities. J. Craniofacial Surg..

[37] Levine D., Barnewolt C.E., Mehta T.S., Trop I., Estroff J., Wong G. (2003). Fetal thoracic abnormalities: MR imaging. Radiology.

[38] Sherwood W., Boyd P., Lakhoo K. (2008). Postnatal outcome of antenatally diagnosed intra-abdominal cysts. Pediatr. Surg. Int..

[39] Hugele F., Dumont C., Boulot P., Couture A., Prodhomme O. (2015). Does prenatal MRI enhance fetal diagnosis of intra-abdominal cysts?. Prenat. Diagn..

[40] Herrera C., Samuel A., Laifer-Narin S., Simpson L., Miller R. (2013). Qualitative performance of fetal MRI compared to ultrasound in cases of multiple fetal anomalies. Am. J. Obstet. Gynecol..

[41] De Wilde J.P., Rivers A.W., Price D.L. (2005). A review of the current use of magnetic resonance imaging in pregnancy and safety implications for the fetus. Prog. Biophys. Mol. Biol..

[42] Abramowicz J.S., Kossoff G., Marsal K., Ter Haar G. (2003). Safety Statement, 2000 (reconfirmed 2003). International Society of Ultrasound in Obstetrics and Gynecology (ISUOG). Ultrasound Obstet. Gynecol..

[43] Griffiths P.D., Bradburn M., Campbell M.J., Cooper C.L., Graham R., Jarvis D., Kilby M.D., Mason G., Mooney C., Robson S.C. (2017). Use of MRI in the diagnosis of fetal brain abnormalities in utero (MERIDIAN): A multicentre, prospective cohort study. Lancet.

[44] Dütemeyer V., Cannie M.M., Badr D.A., Kadji C., Carlin A., Jani J.C. (2023). Prevalence of and risk factors for failure of fetal magnetic resonance imaging due to maternal claustrophobia or malaise. Ultrasound Obstet. Gynecol..

[45] Sohn Y.S., Kim M.J., Kwon J.Y., Kim Y.H., Park Y.W. (2007). The usefulness of fetal MRI for prenatal diagnosis. Yonsei Med. J..

[46] Rossi A.C., Prefumo F. (2014). Additional value of fetal magnetic resonance imaging in the prenatal diagnosis of central nervous system anomalies: A systematic review of the literature. Ultrasound Obstet. Gynecol..

[47] Chauhan N.S., Nandolia K. (2023). Comparison of ultrasound and magnetic resonance imaging findings in evaluation of fetal congenital anomalies: A single-institution prospective observational study. Med. J. Armed Forces India.

